# Resveratrol compares with melatonin in improving in vitro porcine oocyte maturation under heat stress

**DOI:** 10.1186/s40104-016-0093-9

**Published:** 2016-06-03

**Authors:** Yu Li, Jing Wang, Zhenzhen Zhang, Jinyun Yi, Changjiu He, Feng Wang, Xiuzhi Tian, Minghui Yang, Yukun Song, Pingli He, Guoshi Liu

**Affiliations:** National Engineering Laboratory for Animal Breeding, Key Laboratory of Animal Genetics and Breeding of the Ministry of Agriculture, Beijing Key Laboratory for Animal Genetic Improvement, College of Animal Science and Technology, China Agricultural University, Beijing, 100193 China; College of Animal Science and Technology, Jilin Agricultural University, Changchun, Jilin China

**Keywords:** Combination, Maturation Melatonin, Oocyte, Porcine, Resveratrol

## Abstract

**Background:**

Resveratrol, an important phyto-antioxidant commonly found in grapes, mulberry, and other plants, has a variety of functions including anti-aging, anti-cancer and anti-inflammatory activities. In the current study, we investigated the beneficial effects of resveratrol on in vitro porcine oocyte maturation under heat stress (HS). The effect of resveratrol, melatonin and their combination on alleviating HS was compared according to the maturation rate of oocytes and the development competence of embryos after parthenogenetic activation (PA).

**Results:**

Supplementation with resveratrol (2.0 μmol/L) not only improved the nuclear maturation but also raised the blastocyst rate of porcine embryos’ PA from oocytes that underwent HS by increasing their glutathione (GSH) level, reducing reactive oxygen species (ROS) and up-regulating the expression of Sirtuin 1 (*SIRT1*). It was also found that melatonin (10^−7^ mol/L) and the combination of resveratrol (2.0 μmol/L) plus melatonin (10^−7^ mol/L) exhibited more potent effects than resveratrol alone regarding their protective activities on oocyte maturation under HS.

**Conclusions:**

This study compared the efficiencies of resveratrol, melatonin and their combination for protecting porcine oocytes from heat stress. The mechanisms are attributed to the fact that each treatment may have different ability to regulate the synthesis of steroid hormones and the expression of mature related genes.

## Background

Although modern cooling systems are used for preserving porcine, their fertility remains low. A critical issue for their fertility failure is the poor quality of oocyte maturation. HS disrupts the synthesis of the steroid hormone involved in the regulating mechanism of oocyte maturation. Roth et al. found that heat stress retarded the growth of follicles [1] and reduced steroid hormone production, which is related to the low fertility of animals [1–3]. Wolfenson et al. reported that HS caused relatively low plasma concentration of the luteinizing hormone (LH), reduced progesterone secretion by luteal cells and increased the follicle-stimulating hormone (FSH) [4]. The disruption of steroid hormone secretion was caused by the abnormal expression of their synthetic genes in ovaries under heat stress [5]. Ozawa et al. observed that heat stress during the follicular recruitment phase suppressed the subsequent growth to ovulation, accompanied by decreased LH receptor level and estradiol synthesis activity in the follicles [3]. Shimizu et al. reported that heat stress strongly inhibited gonadotropin receptor levels and aromatase activities in granulosa cells. The estradiol levels in the follicular fluid of early antral, antral and preovulatory follicles were also reduced [6]. HS had negative effects on oocyte maturation via down-regulation of the gene expression in steroid hormone synthesis or their receptors. The disruption of the levels of steroid hormones caused the mature failure of in vitro cultured oocytes.

Oxidative stress is another important factor that impedes the cytoplasmic and nuclear maturation of porcine oocyte under HS. Nabenishi et al. reported heat stress on porcine oocyte had an adverse effect on their developmental competence by increasing GSH content and inhibiting the production of oocyte ROS [7]. Similar results were observed by Ozawa et al., who found that heat stress at the zygote stage reduced the developmental ability of mouse embryos via physiological changes in the maternal environment. These changes thus led to an increase in intracellular oxidative stress on the embryo [8]. These phenomena also occurred in bovine oocytes, as reported by Sakatani et al. [9]. An increasing oxidative stress caused by HS in the oviduct was possibly involved in heat stress-induced early embryonic death [10]. HS retarded the nuclear maturation of oocytes, resulting in the poor quality of oocytes, and thus reduced the potency of their development. Most ovine oocytes that matured under heat-shock conditions remained at the germinal vesicle breakdown (GVBD) stage, and they showed an aberrant chromatin configuration in the research of Gharibzadeh [11]. Cherng et al. found that HS caused certain unfavorable changes in the nucleus and cytoskeleton in porcine oocytes, which might be associated with reducing development under the hyperthermic condition and possibly with the low pregnancy rates in domestic species during hot seasons [12]. Payaton et al. observed that heat stress of oocytes during the GV-stage reduced the proportion of oocytes that progressed to metaphase II after in vitro maturation (IVM). Heat treatment for 6 h decreased the proportion of 8- to 16-cell embryos, whereas heat treatment for 12 h reduced blastocyst development [13]. Wang et al. found that spindle assembling was affected and MAD2 was activated in some of the mouse oocytes that matured at 40.7 °C [14].

Resveratrol (3,4,5-trihydroxy-trans-stilbene), an antifungal molecule of the stilbene family, is produced in a variety of plant species, particularly in grapes, in response to pathogen attack or under stressful conditions such as UV radiation [15]. Resveratrol is also a strong antioxidant, maintaining the levels of antioxidant enzymes such as glutathione peroxidase (GPx), superoxide dismutase (SOD), and catalase (CAT) [16, 17] and improving the distribution and function of mitochondria [18]. All these are critical for oocyte maturation. As a phytoestrogen, resveratrol has an equivocal role in regulating the level of estrogen by binding to estrogen receptors (ERs) and evoking biological effects parallel to those exerted by endogenous and synthetic estrogens [19]. It functions as estrogen receptor agonist in different test systems [20]. Resveratrol can enhance the progesterone secretion and expression of luteinization-related genes in the ovary [21]. Many studies have shown that a supplement with resveratrol improves oocyte maturation and the subsequent development in mice [22], bovines [18, 23], goats [24] and porcines [25]. In the current study, the potential effect of resveratrol on in vitro porcine oocyte maturation under HS will be systematically investigated. In addition, its efficiency against HS will be compared with melatonin, another potent antioxidant, and the combination of melatonin with resveratrol.

## Methods

### Chemicals

All chemicals used in this study were purchased from Sigma-Aldrich Co (Alcobendas, Madrid, Spain) unless otherwise indicated.

### Animal studies

All animal studies were conducted in accordance with the requirement of the Institutional Animal Care Committee and have been approved by the Ethics Committee of the China Agriculture University.

#### In vitro maturation

Porcine ovaries were collected from the local abattoir (The Fifth Meat Co, Beijing) and immediately transported in saline (30 ºC) to the laboratory within 4 h. COCs (cumulus-oocyte complexes) were aspirated from the follicles (3–8 mm in diameter) using a 10 mL syringe fixed with an 18-gauge needle. Four or more layers of compact cumulus cells were used for in vitro maturation after washing three times in HEPES-buffered Tyrode’s medium (TLH) containing 0.3 % (w/v) bovine serum albumin (BSA). The COCs were matured (80–90 COCs/500 μL) in the IVM medium (which consisted of TCM199 plus 0.3 % (w/v) BSA containing 10 IU/mL equine chorionic gonadotropin and 10 IU/mL human chorionic gonadotropin, 0.57 mmol/L cysteine and 10 ng/mL epidermal growth factor) at 38.5°Cin 5 % CO_2_.

#### Assessment of polar body rate

To assess the polar body of the metaphase II (MII) oocyte, the samples were collected after 44 h of maturation. The cumulus cells were removed by gently pipetting in the PBS-PVA (0.1 % PVA) medium containing 0.1 % (w/v) hyaluronidase. The oocytes were stained with Hoechst 33342 for 10 min. Then, the samples were rinsed with 0.1 % PVA-DPBS medium three times, and were mounted on a clean glass slide, covered with a coverslip, and examined with an inverted microscope (Nikon Corporation) equipped with epifluorescence.

#### Parthenogenetic activation (PA)

After 44 h of maturation, cumulus cells were removed by gently pipetting in the PBS-PVA (0.1 % PVA) medium containing 0.1 % (w/v) hyaluronidase. Denuded MII oocytes were washed 2–3 times in activation medium (0.28 mol/L mannitol; 0.01 % polyvinyl alcohol; 0.05 mmol/L HEPES; 0.1 mmol/L CaCl_2_ · 2H_2_O and 0.1 mmol/L MgCl_2_) before PA. For activation, an electrical pulse (1.3 KV/cm, 80 μs) generated by a BTX Electro-Cell Manipulator 2001 (BTX, San Diego, CA, USA) was applied to oocytes. After being washed three times with PZM-3 (containing 7.5 μg/mL cytochalasin B(CB) and 10 μg/mL cyclohexane (CHX)), the oocytes were put into PZM-3 (containing 7.5 μg/mL CB and 10 μg/mL CHX, 20 to 30 oocytes/60 μL) medium for 4 h and then washed 3 times with PZM-3. Then, they were cultured for 7 d to evaluate their developmental competence (cleavage rate at d 3 and blastocyst rate at d 7).

#### Measurement of intracellular ROS and GSH levels

The IVM oocytes at the MII stage were sampled 44 h after IVM in a medium with different supplements (resveratrol, melatonin, and their combination) or without to determine their intracellular ROS and GSH levels, as described by Wang et al. [23]. Briefly, 2070-dichlorodihydrofluorescein diacetate (H2DCFDA; Beyotime Institute of Biotechnology, Jiangsu, China) and 4-chloromethyl-6,8-difluoro-7-hydroxycoumarin (Cell Tracker Blue CMF2HC Molecular Probes; Beyotime Institute of Biotechnology) were used to detect intracellular ROS as green fluorescence and the GSH level as blue fluorescence, respectively. A total of 25–30 oocytes from each treatment group were incubated (in the dark) for 30 min in DPBS-PVA containing 10 mmol/L H2DCFDA or 10 mmol/L Cell Tracker Blue. After incubation, the oocytes were washed with DPBS-PVA and placed in 10 μL droplets. Then, the fluorescence was observed using an epifluorescence microscope (TE300; Nikon) with UV filters (460 nm for ROS and 370 nm for GSH). The fluorescent images were saved as graphic files in TIFF format. The fluorescence intensities of the oocytes were analyzed using Image J software (version 1.40; National Institutes of Health, Bethesda, MD) and normalized to that of the control. The experiment was replicated three times.

#### Apoptosis assays (TUNEL) and total cell counts of blastocyst

Apoptosis was detected by terminal deoxynucleotidyl transferase-mediated d-UTP nick end-labeling (TUNEL) assay. Briefly, 7-d-old embryos were washed three times in DPBS-PVP (DPBS supplemented with 0.1 % polyvinylpyrrolidone) and fixed in 4 % (v/v) paraformaldehyde solution for 1 h at room temperature. Membranes were made permeable in 0.1 % Triton X-100 in 0.1 % citrate solution for 1 h at room temperature. Fixed embryos were incubated in TUNEL reaction medium (In Situ Cell Death Detection Kit, Fluorescein; Roche, Mannheim, Germany) for 1 h at 38.5 °C in the dark. Therefore, the broken DNA ends of the embryonic cells were labeled with TdT and fluorescein-dUTP. After the reaction stopped, the embryos were washed in DPBS-PVA and mounted on glass slides with DAKO Fluorescent Mounting Medium (S3023, Dako North America, Carpinteria, CA, USA) containing Hoechst 33342 for total cell counts. Whole-mount embryos were examined under an epifluorescence microscope (Nikon) by TUNEL assay and Hoechst staining. The numbers of apoptotic nuclei (by TUNEL assay) and the total numbers of nuclei were determined from optical images. The apoptotic rate was calculated as follows: apoptotic rate = (number of TUNEL-positive nuclei/total number of nuclei) × 100 %.

#### RNA isolation and quantitative real-time polymerase chain reaction

Fresh immature COCs were collected after HS, washed twice with DPBS solution and stored at −80 °C until the RNA was extracted. The total RNA was extracted using TRIzol reagent (Invitrogen Inc., Carlsbad, CA, USA), quantified by measuring the absorbance at 260 nm and stored at −80 °C until use. The levels of relevant mRNA, including the products of antioxidant-related genes, apoptosis-related genes and maturation-related genes, were determined by quantitative real-time polymerase chain reaction (RT-PCR) using a One Step SYBR Prime Script RT-PCR Kit (TaKaRa Bio Inc., Tokyo, Japan) in a Light Cycler instrument (Roche, Mannheim, Germany). The levels of accumulated fluorescence were analyzed using the second derivative method after the melting-curve analysis was complete. Then, the expression levels of the target genes were normalized to the expression level of actin in each sample. The primer pairs for the analyzed mRNAs are listed in Table 1.

**Table 1:** Primers used in this study

Gene	Primer sequence*(5’-3*’)	*T*_m_(°C)	Size,bp
*β-actin*	Forward: GTGGACATCAGGAAGGACCTCTA Reverse: ATGATCTTGATCTTCATGGTGCT	60.0	131
*HSP70*	Forward: TTCGACGTGTCCATCCTGACG Reverse: TCACCGCCCGCTTGTTCTGG	60.0	169
*Sphk1*	Forward: CCAGGTGCACCCAAACTACT Reverse: GAGGCTTCTGGCTGAGTGAG	60.0	92
*SOD1*	Forward: TCCATGTCCATCAGTTTGGA Reverse: AGTCACATTGGCCCAGGTCTC	60.0	131
*CYP11A*	Forward:TGGTGACAATGGCTGGATTAACCT Reverse: GACGAAGTCCTGAGACACTGTGT	60.0	376
*CYP19*	Forward: GTCGTGGACTTCGTCATG Reverse: AGTGTGACCAAGATGACCTT	60.0	257
*SIRT1*	Forward: TTGATCTTCTCATTGTTATTGGGTC Reverse: ACTTGGAATTAGTGCTACTGGTCTTA	60.0	62
*Akt2*	Forward: AAAGTCATCCTGGTGCG Reverse: GGGTGCCTGGTGTTCTG	60.0	136

### Experimental procedures

All experiments were conducted in a heat-stress model (42 °C, 20–24 h IVM) chosen from different HS strategies, which can significantly reduce the polar body rate of oocytes and the blastocyte rate of porcine PA embryos [26]. Resveratrol (0.5, 2.0, 5.0, and 10.0 μmol/L), melatonin (10^−7^ mol/L), which was set according to the previous study [26], and the combination of melatonin (10^−7^ mol/L) and resveratrol (2.0 μmol/L) were added to the maturation solution. The intracellular levels of ROS and GSH, the total number of blastocysts, the apoptosis cell rate in blastocysts, and the expression of genes associated with oxidation apoptosis maturation were tested to compare the efficiency of resveratrol and melatonin and their combination to protect porcine oocyte from HS.

### Statistics analysis

Data are expressed as the mean ± S.E.M. Statistical analyses were performed using univariate analysis of variance (ANOVA) with the aid of SPSS 19.0 statistical software, followed by Student’s *t*-test. *P* < *0.05* was considered significant, and *P* < *0.01* was considered highly significant.

## Results

### Effect of resveratrol on maturation of porcine oocytes under heat stress

**Fig. 1:**
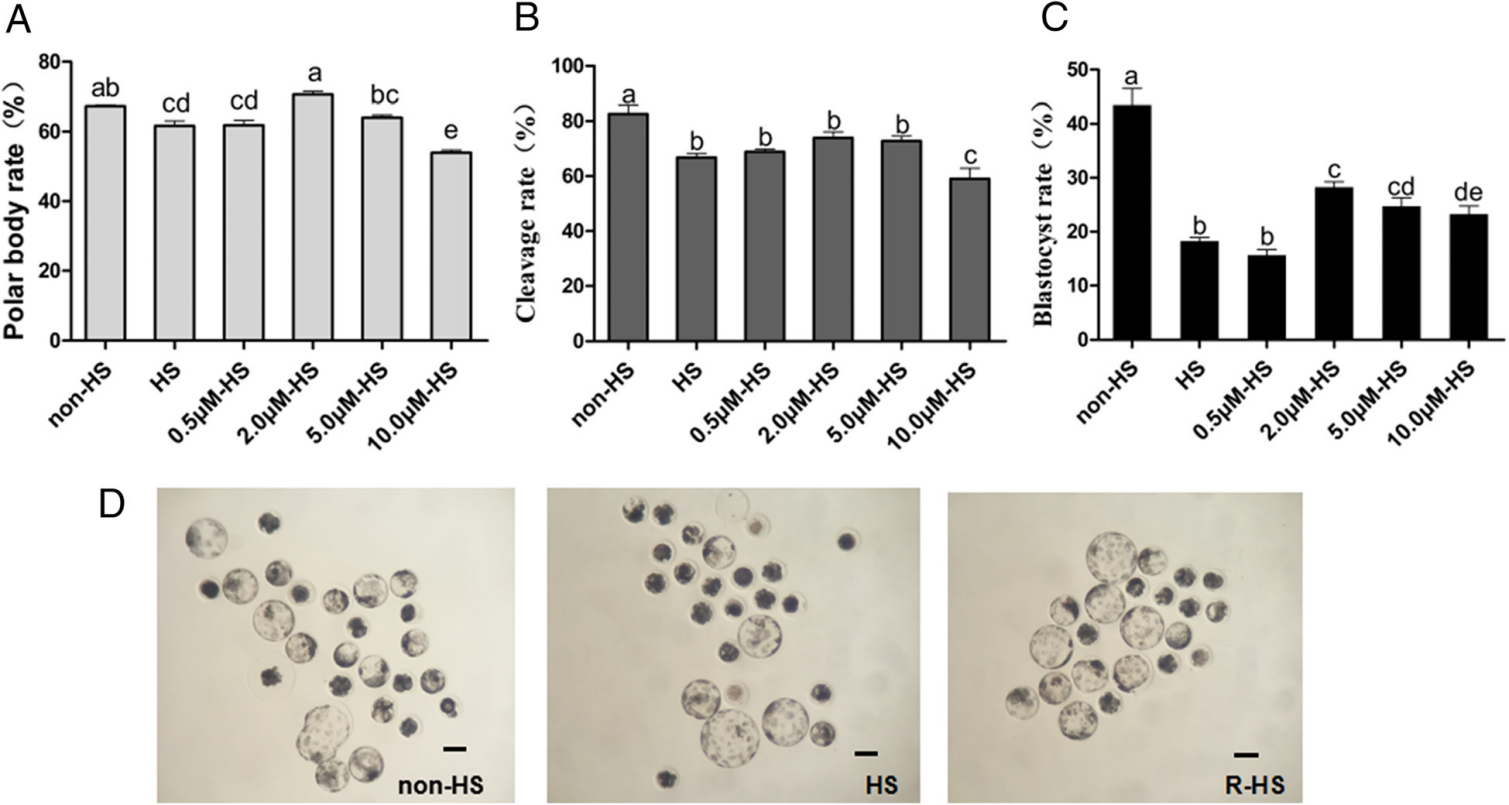
Effect of different concentrations of resveratrol on the PB1 rate of porcine oocyte and development competence after PA. **a** Effect of different concentrations of resveratrol on the PB rate. **b** Effect of different concentrations of resveratrol on the cleavage rate of porcine PA embryo. **c** Effect of different concentrations of resveratrol on the blastocyst rate of porcine PA embryo. **d** Blastocyst from non-heat stress, heat stress and resveratrol (2.0 μmol/L). bar = 100 μm. non-HS: non-heat stress; HS: heat stress; R:resvertrol

A total of 1098 oocytes (more than six replications) underwent maturation with or without heat treatment with different concentrations of resveratrol (0.5, 2.0, 5.0, and 10.0 μmol/L). After maturation (IVM 44 h), the polar body rates were tested. The results showed that the polar body rate of oocytes that underwent heat stress (HS group) was significantly lower than that of the non-HS group (67.32 ± 0.30 % and 61.62 ± 1.39 %, respectively; *P* < *0.05*). The polar body rate at the 2.0 μmol/L resveratrol (R) group was significantly higher (70.62 ± 0.87, *P* < *0.05*), and there were no obvious changes in the 0.5 and 5.0 μmol/L resveratrol-treated groups (61.82 ± 1.41 % and 64.01 ± 0.76 %; *P* > *0.05*). In contrast, the 10.0 μmol/L resveratrol group had the lowest polar body rate (53.95 ± 0.01 %; Fig. 1a).

After PA, the cleavage rate of embryos from oocytes with heat treatment was significantly lower than that of embryos from the non-HS group (76.78 ± 1.49 % *vs* 82.64 ± 3.18 %; *P* < *0.05*). The cleavage rate of resveratrol-treated groups failed to increase (68.88 ± 1.01 %, 73.92 ± 8.38 %, and 72.77 ± 6.86; *P* > *0.05*; Fig. 1b). On the other hand, heat stress significantly reduced the blastocyst rate of embryos after PA (18.28 ± 0.66 % *vs* 43.48 ± 3.07 %; *P* < *0.05*), and the supplement with 2.0, 5.0, and 10.0 μmol/L resveratrol enhanced the blastocyst rate significantly (28.27 ± 1.02 %, 24.75 ± 1.52 %, and 23.25 ± 1.50 %; *P* < *0.05*; Fig. 1c).

### Effects of resveratrol, melatonin and their combination on maturation of porcine oocyte under heat stress

**Fig. 2:**
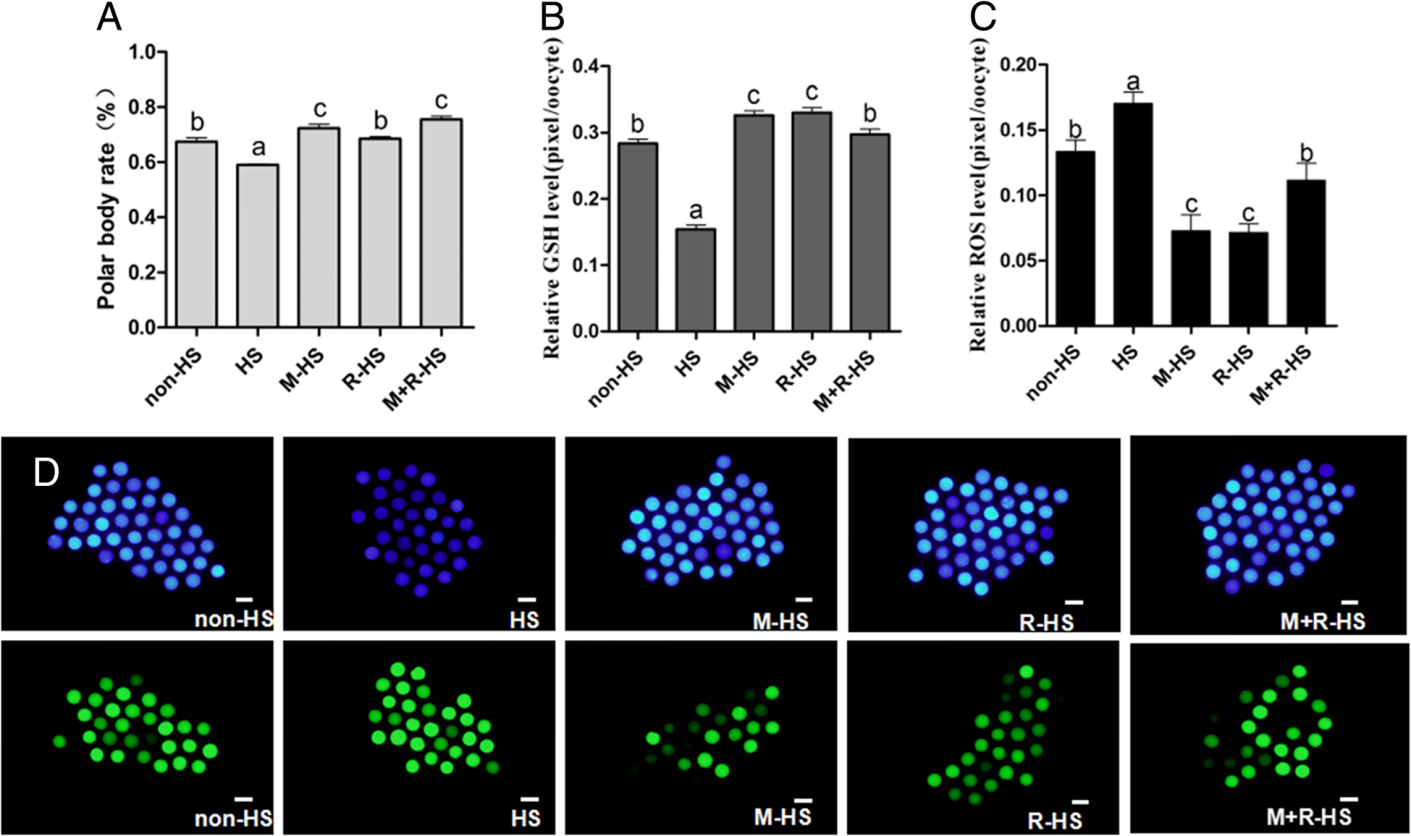
Effect of different supplements on the maturation of porcine oocytes under HS. **a** Effect of different supplements on the PB1 rate of porcine oocyte. **b** Effect of different supplements on the GSH level. **c** Effect of different supplements on the ROS level. **d** Effect of different supplements on the ROS and GSH levels. non-HS: non-heat stress; HS: heat stress; M-HS: supplement melatonin under heat stress; R-HS: supplement resveratrol under heat stress; M + R-HS: under heat stress melatonin and resveratrol under heat stress

Melatonin (10^−7^ mol/L), resveratrol (2.0 μmol/L) and their combination were selected to compare their ability against heat stress. The results showed that the polar body rates of the melatonin group (M-HS) and the combination group (M + R-HS) were significantly higher than those of the non-HS group (72.37 ± 1.44 % and 75.57 ± 1.17 % *vs* 67.47 ± 1.44 %; *P* < *0.05*). The polar body rate of the resveratrol group (R-HS) was significantly higher than that of the HS group (68.53 ± 0.75 % *vs* 59.08 ± 0.31 %; *P* < *0.05*). The data indicated that resveratrol, melatonin and their combination could improve the polar body rate, which was reduced by heat stress, and the effects of melatonin and the combination on the polar body rate were significantly higher than those of resveratrol alone (Fig. 2a).

The intracellular levels of GSH in the oocytes in the melatonin and resveratrol groups were significantly higher than those in the non-HS group (0.3262 ± 0.0071 and 0.3302 ± 0.0076 pixels/oocytes *vs* 0.2837 ± 0.0069 pixels/oocytes, respectively; *P* < *0.05*). The GSH level of the oocytes in the combination group was lower than the GSH level of those in melatonin or resveratrol alone groups; however, it was still significantly higher than that in the heat stress group (0.2975 ± 0.0079 pixels/oocytes *vs* 0.1543 ± 0.0062 pixels/oocytes; *P* < *0.05*) and similar to the level of the non-HS group (Fig. 2b). Consistent with this finding, the ROS level was significantly lower in the melatonin- and resveratrol-treated samples than that in non-HS groups (0.0728 ± 0.0124 and 0.0715 ± 0.0067 pixels/oocytes *vs* 0.1336 ± 0.0091 pixels/oocytes, respectively; *P* < *0.05*). The ROS level in the combination group was also significantly lower than that in heat stress group (0.1118 ± 0.0129 pixels/oocytes *vs* 0.1706 ± 0.0087 pixels/oocytes; *P* < 0.05). These data indicated that all the treatments of resveratrol, melatonin and their combination could help to relieve oocytes from heat stress. The effect of either melatonin or resveratrol was significantly higher than their combination (Fig. 2c).

### Comparison of different treatments on the equality of PA embryo

**Fig. 3:**
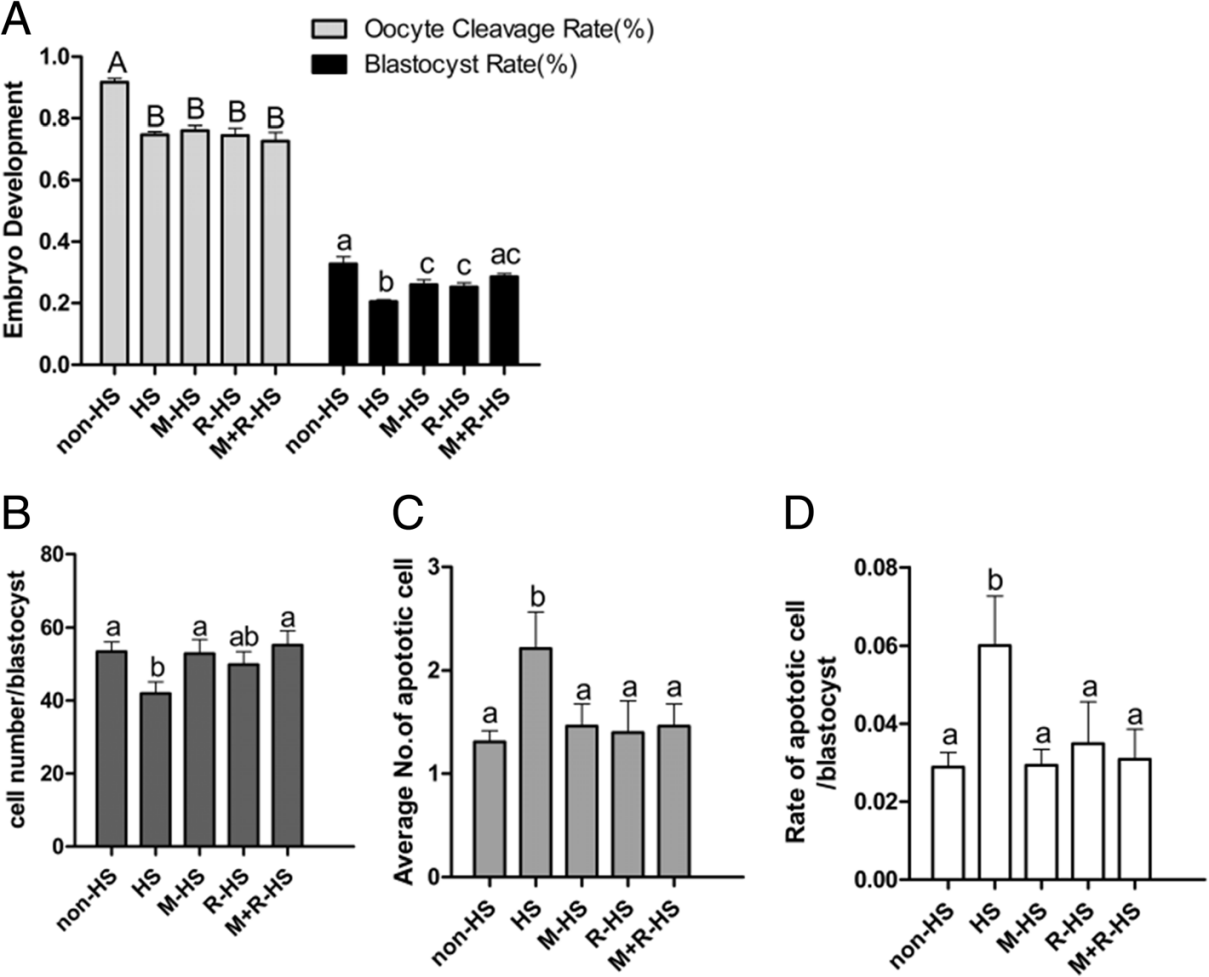
Effect of different supplements on the quality of PA embryo from oocyte under HS. **a** Effect of different supplements on the development competence of PA embryo from oocyte under HS. **b** Effect of different supplements on the cell number of blastocysts. **c** Effect of different supplements on the apoptotic cell number of blastocysts. **d** Effect of different supplements on the apoptotic cell rate of blastocysts. non-HS: non-heat stress; HS: heat stress; M-HS: supplement melatonin under heat stress; R-HS: supplement resveratrol under heat stress; M + R-HS: under heat stress melatonin and resveratrol under heat stress

After PA, the cleavage rates of embryos from oocytes supplemented with melatonin, resveratrol and their combination had no significant changes compared with the HS group (76.11 ± 1.58 %, 74.57 ± 2.32 %, and 72.69 ± 2.81 % *vs* 74.75 ± 1.04 %, respectively; *P* > *0.05*). The cleavage rates were significantly lower than those of embryos from the non-HS group (91.67 ± 1.31 %, *P* < *0.05*). However, heat stress significantly reduced the blastocyst rate of embryos after PA (20.51 ± 0.58 % *vs* 32.77 ± 2.33 %; *P* < *0.05*), and the supplementations of melatonin, resveratrol and their combination raised the blastocyst rate significantly (26.03 ± 1.63 %, 25.32 ± 1.33 %, and 28.66 ± 0.94 %, respectively; *P* < *0.05*). The blastocyst rate in the combination group was not significantly different from that of the non-HS group (*P* > *0.05*). The combination of melatonin and resveratrol was the most effective treatment regarding the increasing blastocyst rate under the HS (Fig. 3a).

Heat stress significantly reduced the total cell number of blastocysts compared to the non-HS group (41.96 ± 3.17 *vs* 53.44 ± 2.65; *P* < *0.05*), whereas the melatonin and combination groups preserved the total cell number at a similar level to the non-HS group (52.90 ± 3.84 and 55.26 ± 3.91; *P* < *0.05*). The total cell number of blastocysts in the resveratrol group (49.9 ± 3.56) was not significantly different from that of the non-HS group or the heat stress group (*P* > 0.05; Fig. 3b). However, the heat stress group had a significantly higher apoptosis cell number than the non-HS group (2.21 ± 0.35 *vs* 1.31 ± 0.11; *P* < *0.05*). The melatonin, resveratrol and combination groups significantly reduced apoptotic cells (1.46 ± 0.22, 1.40 ± 0.31, and 1.46 ± 0.22, respectively; *P* < *0.05*; Fig. 3c). The apoptotic cell rate showed a similar tendency to the apoptotic cells in different treated groups (6.00 ± 1.26 % *vs* 2.88 ± 0.38 %, 2.93 ± 0.41 %, 3.48 ± 1.07 % and 3.08 ± 0.78 %, respectively; *P* < *0.05*; Fig. 3d).

### Different supplements on the expression of genes in COCs

**Fig. 4:**
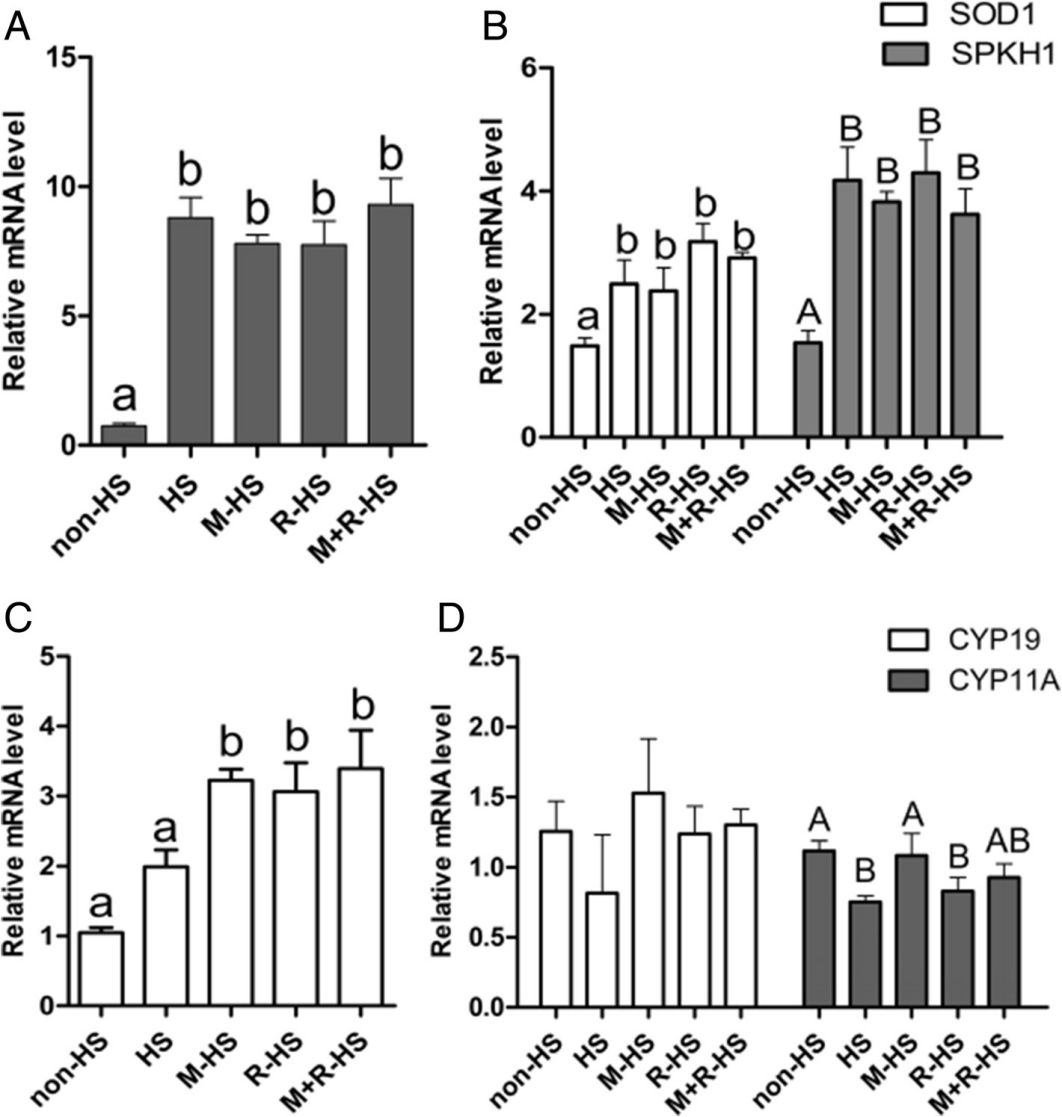
Effect of different supplements on the expression of genes. **a** Effect of different supplements on the expression of HSP70. **b** Effect of different supplements on the expression of genes associated with antioxidants and apoptosis. **c** Effect of different supplements on the expression of SIRT1. **d** Effect of different supplements on the expression of genes associated with the synthesis of steroid hormones. non-HS: non-heat stress; HS: heat stress; M-HS: supplement melatonin under heat stress; R-HS: supplement resveratrol under heat stress; M + R-HS: under heat stress melatonin and resveratrol under heat stress

The level of *HSP70* gene expressed in the COCs was significantly higher in the heat-treatment group than that in the non-HS group (*P* < *0.05*). Melatonin, resveratrol and their combination did not significantly affect the *HSP70* gene expression when compared with HS group (*P* > *0.05*; Fig. 4a). Regarding antioxidant-related gene *SOD1* and apoptosis-related gene *SPKH1*, heat stress significantly enhanced their expressions (*P* < *0.05*), whereas treatments with melatonin, resveratrol or their combination did not modify the expression levels of these genes, which were up-regulated by HS (*P* > *0.05*; Fig. 4b). Melatonin, resveratrol and their combination significantly raised the expression of *SIRT1* compared with the non-HS and HS group (*P* < *0.05*; Fig. 4c). As to the genes involved in steroidogenesis, *CYP11A* and *CYP19* were reported to be expressed in cumulus cells and take part in the synthesis of progesterone and estrogen, respectively [27, 28]. Heat stress reduced the expression of *CYP11A*. The melatonin and combination groups showed strong abilities in up-regulating the expression of *CYP11A* to the level of the non-HS group, whereas resveratrol had no significant effect in up-regulating the expression of this gene (*P* > *0.05*). All treatments had no significant effects on the expression of *CYP19* (*P* > *0.05*; Fig. 4d).

## Discussion

In the current study, we found that the polar body rate, an index of the nucleus maturation of porcine oocytes, was retarded by heat stress. Resveratrol (2.0 μmol/L) relieved the adverse effect and increased the proportion of oocytes that had matured into the MII stage (Fig. 1a). This effect of resveratrol may attribute its ability to be an antioxidant. Resveratrol has a strong capacity to reduce oxidant damage [29]. It improves the maturation environment by alleviating the high level of ROS in heat-treated porcine oocytes. Our results confirmed that resveratrol (2.0 μmol/L) not only scavenged ROS but also improved the intracellular GSH level of oocytes to further fight oxidative stress (Fig. 2b-c). This finding was consistent with the result of Kwak et al. [25]. These authors reported that resveratrol failed to increase the polar body rate of porcine oocytes in the normal case. However, our data showed that resveratrol (2.0 μmol/L) improved nuclear maturation, which indicated resveratrol had a direct effect for protecting porcine oocytes from HS by reducing the high level of ROS and helping them produce more GSH. In a word, it provided a proper environment for nuclear maturation. We also found that resveratrol (2.0 μmol/L) enhanced the expression of *SIRT1* (Fig. 4c), which had been observed in cows [23] and mice [22]. *SIRT1* was reported to associate with regulating autophagy and mitochondrial function in cells upon oxidative stress [30]. Resveratrol was found to improve mitochondrial function by activating *SIRT1* [31, 32]. This finding was confirmed in the current study; i.e., the expression of *SIRT1* was up-regulated by resveratrol under HS. Our data indicated that resveratrol (2.0 μmol/L) promoted the nucleus maturation of porcine oocytes by reducing the oxidant stress caused by heat, and it ensured cytoplasm maturation by improving the function of mitochondria through *SIRT1*.

Resveratrol (2.0 μmol/L) promoted the development competence of embryos PA from oocytes that underwent HS with a higher blastocyst rate (Fig. 1c) and quality (Fig. 3b, c, d), which was also reported in bovines [23], goats [24], mice [22], and porcines [25]. Resveratrol prevented cells from apoptosis (Fig. 3c-d). Kong et al. reported that resveratrol delayed oocyte nest breakdown and inhibited both the primordial-to-developing follicle transition and apoptosis by decreasing the activation of *Foxo3a*, *Bim*, and *p27KIP1* [33]. Bucci et al. observed that resveratrol modulated the porcine oocyte apoptotic process caused by cryopreservation-induced damage [34]. Notably, resveratrol didn’t show dose effect. The 2.0 μmol/L was the most efficient concentration that protected the porcine oocyte from HS during maturation. Resveratrol of other concentrations showed little or even an adverse effect. The same results were observed by Wang et al.[23]. They found that substantially high concentrations of resveratrol might have a negative effect on bovine oocyte maturation, which might be caused by the competitive inhibition of the activities of various phosphodiesterases, resulting in an increase in the concentration of cytosolic cAMP. By contrast, 10.0 μmol/L resveratrol failed to regulate the synthesis of steroid hormones, ultimately lead to retarding oocyte maturation [23, 35].

There were few studies to compare the efficiency of melatonin and resveratrol on the oocyte maturation under the in vitro condition. Ebly et al. showed that melatonin was more potent than resveratrol in promoting the expression and activity of GSH and glutathione peroxidase in liver, whereas in the activation of liver catalase, resveratrol was stronger than melatonin [36]. López et al. [37] compared the effect of melatonin and other antioxidants in reducing DNA damage. They found that 100 μmol/L resveratrol could reduce 78 ± 4 % 8-OH-dG (an indicator of cell DNA damage), but the IC50 (half inhibitory concentration) of resveratrol was 10.9 ± 0.3 μmol/L, which is three times higher than that of melatonin. They also found that melatonin could eliminate the oxidation damage produced by low doses of resveratrol, and supplementation with melatonin and resveratrol together did not show a synergistic effect on DNA protection. Our results also showed that their combination, despite significantly reducing the level of ROS and increasing the level of GSH compared with the heat stress group, was significant weaker in changing the level of ROS and GSH than supplementation with melatonin or resveratrol alone (Fig. 2b-c).

Melatonin can work synergistically with vitamin C [37]. Other research showed that melatonin can strengthen the nerve protective effect of resveratrol by inhibiting protease ubiquitin-proteasome, which can promote hemoglobin oxidase to ease some neurodegenerative diseases [38]. In the current study, we observed that melatonin was more potent than resveratrol in increasing the polar body rate under the condition of HS (Fig. 2a). This finding was consistent with the observations of others [36, 37]. The potential mechanisms may contribute to melatonin’s favorable distribution in both the lipid and water phases [39] and its cascade reaction with free radicals [40]. The significantly weaker ability of resveratrol in comparison to melatonin to up-regulate the expression of *CYP11A* under heat stress could be another reason (Fig. 4d). As we all know, melatonin is a hormone that can be synthesized in mammals, and its receptors are found in many organs, tissues, and cells, such as oocyte [41]. Melatonin functions through its receptors, for example, promoting bovine embryo development in vitro by MT1 [32]. By contrast, resveratrol is merely a type of phytohormone without a receptor in animals. We also observed the synergistic effect of melatonin and resveratrol in terms of their protective effects on in vitro porcine oocyte maturation under HS. However, similarly to the melatonin and resveratrol groups, the combination showed no significant improvement in the cleavage rate (Fig. 3a). It increased the blastocyst rate, the endpoint for the in vitro development of embryos, under HS at the greatest extent compared with melatonin and resveratrol alone (Fig. 3a), showing no significant difference from the non-HS group. This may have been caused by the right ability combination to balance the ROS and GSH to the levels of the non-HS group, as shown in Fig. 2b-c. As we all know, ROS takes part in some physiological pathway, such as activating the cascade signal in cells [42]. Therefore, a proper level of ROS was needed. The large rate of GSH/ROS when supplement melatonin or resveratrol alone may have caused the final failure to increase the blastocyst rate to the level of the non-HS group, whereas the combination group achieved it. These results provide valuable information regarding how to use well-known antioxidants to protect oocytes from oxidative stress under unfavorable conditions, including the HS and in vitro maturation. It seems that the different maturation stages of oocytes require different antioxidant strategies. For example, in certain maturation stages, melatonin or resveratrol alone provides better protection, and in other stages, their combination is more suitable. These will be our future projects of investigation.

## Conclusions

Resveratrol (2.0 μmol/L) protected porcine oocyte maturation in vitro from heat stress (IVM 20–24 h, 42 °C) by significantly eliminating ROS, increasing GSH, and up-regulating the expression of *SIRT1*. Comparatively, melatonin (10^−7^ mol/L) and the combination were more potent in alleviating the adverse effects of HS on the polar body rate than was resveratrol alone. With the combined treatment, the polar body rate was even higher than that of the controls. Melatonin, resveratrol and their combination failed to improve the cleavage rate of the PA embryo, but they raised the blastocyst rate compared to the group under HS, and the combination group had the strongest ability in this respect. Melatonin and the combination increased the total cell number of blastocysts to a higher level than resveratrol did, and they all effectively reduced apoptosis under HS. Generally, the combination was stronger than melatonin in the efficiency of alleviating the adverse effect of heat stress on porcine oocyte, and melatonin was stronger than resveratrol.
